# Plasma microRNAs levels are different between pulmonary and extrapulmonary ARDS patients: a clinical observational study

**DOI:** 10.1186/s13613-018-0370-1

**Published:** 2018-02-13

**Authors:** Yi Zheng, Song-qiao Liu, Qin Sun, Jian-feng Xie, Jing-yuan Xu, Qing Li, Chun Pan, Ling Liu, Ying-zi Huang

**Affiliations:** 1 Department of Critical Care Medicine, Zhongda Hospital, School of Medicine, Southeast University, No. 87, Dingjiaqiao Road, Gulou District, Nanjing, 210009 China; 2 Department of Critical Care Medicine, The First Affiliated Hospital of Medical School of Zhejiang University, 79 Qingchun Road, Shangcheng District, Hangzhou, 310003 China

**Keywords:** Pulmonary ARDS, Extrapulmonary ARDS, MicroRNA, Vascular endothelial cell

## Abstract

**Background:**

Mesenchymal stem cells (MSC) obviously alleviate the damage of the structure and function of pulmonary vascular endothelial cells (VEC). The therapeutic effects of MSC are significantly different between pulmonary ARDS (ARDSp) and extrapulmonary ARDS (ARDSexp). MicroRNAs (miRNAs), as important media of MSC regulating VEC, are not studied between ARDSp and ARDSexp. We aimed to explore the plasma levels difference of miRNAs that regulate VEC function and are associated with MSC (*MSC*-*VEC*-miRNAs) between ARDSp and ARDSexp patients.

**Methods:**

*MSC*-*VEC*-miRNAs were obtained through reviewing relevant literatures screened in PubMed database. We enrolled 57 ARDS patients within 24 h of admission to the ICU and then collected blood samples, extracted plasma supernatant. Patients’ clinical data were collected. Then, plasma expression of *MSC*-*VEC*-miRNAs was measured by real-time fluorescence quantitative PCR. Simultaneously, plasma endothelial injury markers VCAM-1, vWF and inflammatory factors TNF-α, IL-10 were detected by ELISA method.

**Results:**

Fourteen miRNAs were picked out after screening. A total of 57 ARDS patients were included in this study, among which 43 cases pertained to ARDSp group and 14 cases pertained to ARDSexp group. Plasma miR-221 and miR-27b levels in ARDSexp group exhibited significantly lower than that in ARDSp group (miR-221, 0.22 [0.12–0.49] vs. 0.57 [0.22–1.57], *P* = 0.008, miR-27b, 0.34 [0.10–0.46] vs. 0.60 [0.20–1.46], *P* = 0.025). Plasma vWF concentration in ARDSexp group exhibited significantly lower than that in ARDSp group (0.77 [0.29–1.54] vs. 1.80 [0.95–3.51], *P* = 0.048). Significant positive correlation was found between miR-221 and vWF in plasma levels (*r* = 0.688, *P* = 0.022). Plasma miR-26a and miR-27a levels in non-survival group exhibited significantly lower than that in survival group (miR-26a, 0.17 [0.08–0.20] vs. 0.69 [0.24–2.33] *P* = 0.018, miR-27a, 0.23 [0.16–0.58] vs. 1.45 [0.38–3.63], *P* = 0.021) in ARDSp patients.

**Conclusion:**

Plasma miR-221, miR-27b and vWF levels in ARDSexp group are significantly lower than that in ARDSp group. Plasma miR-26a and miR-27a levels in non-survival group are significantly lower than that in survival group in ARDSp patients.

**Electronic supplementary material:**

The online version of this article (10.1186/s13613-018-0370-1) contains supplementary material, which is available to authorized users.

## Background

Acute respiratory distress syndrome (ARDS) is a common critical disease in intensive care unit (ICU). In recent years, although mechanical ventilation, liquid management, extracorporeal membrane oxygenation and other therapeutic technologies have improved significantly, ARDS is associated with high morbidity and mortality in critically ill patients [1]. Endothelial dysfunction is a key characteristic of ARDS, giving rise to increasing vascular permeability and then pulmonary edema and respiratory failure [2, 3]. The biological underpinnings manipulating the development of endothelial dysfunction in ARDS are incompletely cognized and represent the inevitable course to precision diagnosis and treatment.

Patients with ARDS which is a heterogeneous syndrome have variant etiologies and pathologies and respond differently to therapeutic interventions [4]. One approach to reducing ARDS heterogeneity is to subclassify patients as ARDSp (originating from pulmonary disease) or ARDSexp (originating from extrapulmonary disease) [5]. In the early stages of ARDS, there were significant differences in damage degree of endothelial cells between ARDSp and ARDSexp [6, 7]. When lung morphology was analyzed by computed tomography (CT), ARDSp was characterized by prominent consolidation, while ARDSexp was characterized by prominent ground-glass opacification [8]. The two subtypes of ARDS respond differently to therapeutic interventions such as alterations in positive end-expiratory pressure, prone ventilation, and recruitment maneuvers [9–13]. Nevertheless, the underlying mechanism governing this difference needs further research.

Mesenchymal stem cells (MSC), protecting adherens junction (VE-cadherin and β-catenin), reducing the lung endothelial cell apoptosis, improve pulmonary vascular endothelial cells (VEC) permeability of ARDS [14–18]. However, the therapeutic effects of MSC are significantly different between ARDSp and ARDSexp [19]. This is similar to bone marrow-derived mononuclear cell more effectively improving survival, lung mechanics and histology in ARDSexp than these in ARDSp [20]. The mechanism of difference is not entirely clear.

MicroRNAs (miRNAs), a group of small (19–25 nucleotides) non-coding segments of RNA, regulate gene expression by binding to target mRNA to inhibit their translation. MiRNAs also play an important role in the regulation of gene expression in the pathogenesis of ARDS. Previous studies [21, 22] showed that MSC control activity of pulmonary VEC through regulating microRNAs (miRNAs) levels. Herein, we tentatively defined *MSC*-*VEC*-miRNAs as a group of miRNAs which are associated with MSC, have regulatory effects on VEC and have previously been studied in ARDS. Then, levels of *MSC*-*VEC*-miRNAs can be different in patients with ARDSp and ARDSexp.

Yet, so far, no study has tested whether *MSC*-*VEC*-miRNAs may serve as biomarkers distinguish between ARDSp and ARDSexp. In this study, 14 *MSC*-*VEC*-miRNAs were filtrated through relevant literatures. Further, we have examined the expression levels of these *MSC*-*VEC*-miRNAs in plasma collected from patients diagnosed as ARDSp and ARDSexp. Our purpose is to explore the plasma levels difference of *MSC*-*VEC*-miRNAs between ARDSp and ARDSexp which is probably helpful for the study in pathogenesis and clinical diagnosis of ARDSp and ARDSexp.

## Methods

### Screening of *MSC*-*VEC*-miRNA

Using the combination of keywords and MeSH terms for “endothelial cell” and “microRNA”, we searched PubMed for articles that describe associations between the miRNAs and endothelial cell. Each article was reviewed and associated miRNAs (“miRNAs cluster 1”) were recorded. Then, we searched each miRNA in “miRNAs cluster 1” individually in conjunction with mesenchymal stromal cell (e.g., “miR-21” and “mesenchymal stromal cell”) and reviewed each article to get miRNAs (“miRNAs cluster 2”) associated with mesenchymal stromal cell from “miRNAs cluster 1”. Using the same method, we obtained *MSC*-*VEC*-miRNAs, eligible microRNAs that were associated with MSC, has regulatory effects on VEC and has previously been studied in ARDS (Additional file 1: Table S1).

### Subject recruitment and sample acquisition

All new ICU admissions at Zhongda Hospital Affiliated to Southeast University from January 2016 to September 2016 were screened for the presence of ARDS based on acute respiratory distress syndrome: the Berlin Definition [23]. Additional inclusion criteria included 18 years ≤ age ≤ 89 years and admission into the ICU within the previous 24 h. We excluded immunocompromised patients including history of stem cell transplant, immunosuppressive medication using and excluded patients with malignant tumor and pregnant women.

After signing informed consent, subjects had blood drawn via venipuncture or from pre-existing intravascular catheters. Blood samples from enrolled patients were obtained within 24 h of admission to the ICU. Samples were centrifuged at 1900*g* for 10 min, and the plasma supernatant was extracted and stored in refrigeratory at − 80 degrees Celsius.

### Patients data collection

Demographic and clinical data from eligible patients was abstracted from the electronic medical record. Demographic data: gender, age, actual height, actual weight, etc. Patient’s condition: main diagnosis, acute physiology and chronic health evaluation (APACHE) II scores, sequential organ failure assessment (SOFA) scores, ARDS etiology. ARDS severity: arterial blood PO_2_/FiO_2_ ratio, Murray lung injury score. The style of oxygen therapy and parameters: noninvasive ventilation, invasive ventilation and ventilator parameters. Clinical outcomes: ICU and hospital length of stay, 28-day mortality, occurrence of shock (defined by clinician), occurrence of acute kidney injury [KDIGO Clinical Practice Guideline for Acute Kidney Injury].

### RNA isolation

The frozen plasma was taken out from refrigeratory and incubated at 37 °C in a water bath until samples are completely thawed. Prolonged incubation should be avoided, which may compromise RNA integrity. RNAs were isolated from plasma samples using miRNeasy serum/plasma kits (Qiagen). The miRNeasy Serum/Plasma Spike-In Control, a *Caenorhabditis elegans* miR-39 miRNA mimic, was chosen as the normalized internal control. 3.5 μl miRNeasy Serum/Plasma Spike-In Control (1.6 × 10^8^ copies/μl working solution) was added to the tube containing the lysate before adding chloroform in the RNA extraction process.

### Real-time PCR

After total RNA isolation, quantitative real-time PCR (qRT-PCR) was performed with a miScript System (Qiagen, USA). All procedures were performed according to the instructions provided by the manufacturer. Reverse transcription (RT) was done in a reaction component of 20 μl, which contained 2 μl miScript Reverse Transcriptase Mix, 2 μl miScript Nucleics Mix, 4 μl miScript HiSpec Buffer, a certain volume of template RNA containing 100 ng total RNA and a little RNase-free water increasing reaction volume to 20 μl. The mixture was incubated 37 °C for 60 min and 95 °C for 5 min. The 20 μl RT product was diluted into 100 μl. Reaction system of quantitative real-time PCR contained 10 μl SYBR Green PCR Master Mix, 2 μl miScript specific primer, 2 μl miScript universal primer, 2 μl cDNA and 4 μl RNase-free water. qRT-PCR used an Applied Biosystems StepOne detection system at 95 °C for 15 min, followed by 40 cycles of 95 °C for 15 s, 55 °C for 30 s, 70 °C for 30 s. All qRT-PCRs were performed in triplicate, and the raw Ct (threshold cycle) of each sample was the mean value of three Ct values. The data were analyzed by the 2^−ΔΔCT^ method.

### Statistical analysis

Baseline characteristics and clinical condition indicator of human subjects were compared between ARDSp and ARDSexp. Expression levels of selected miRNAs detected by qRT-PCR were normalized to miR-39 and analyzed using the 2^−ΔΔCT^ method. Results for normally distributed continuous variables are presented as mean ± SD and compared between groups by Student’s *t* tests. Results for non-normally distributed continuous variables are summarized as medians [interquartile ranges] and were compared by Mann–Whitney *U* tests. Results for categorical variables are presented as sample rate (constituent ratio) and were compared Chi-squared test or Fisher exact test. Logistic regression analysis was carried out to determine the variables that were associated independently with the death of ARDSp patients. We examined whether miR-26a and miR-27a were independent risk factors for the death after adjustment for age and APACHE II score. All tests were two-sided, and *P* values < 0.05 were considered statistically significant.

## Results

### Screening result of *MSC*-*VEC*-miRNA

Fourteen miRNAs were picked out which include miR-15a, miR-16, miR-21, miR-24, miR-26a, miR-27a, miR-27b, miR-126, miR-146a, miR-150, miR-155, miR-221, miR-223, miR-320. Relevant references were presented with PubMed Unique Identifier in Additional file 2: Table S2. The detail information of these miRNAs is shown in Table 1.

**Table 1:** Summary of candidate *MSC*-*VEC*-miRNAs Regulation in vascular endothelial cells

MiRNAs	Function on angiogenic process	Gene targets	Adjusting direction
miR-15a	Inhibits angiogenesis through direct targeting of VEGF and FGF	FGF2, FGFR1, VEGF, VEGFR2	–
miR-16	Inhibits tumor angiogenesis and EC-mediated angiogenesis in vitro and in vivo	FGF2, FGFR1, VEGF, VEGFR2	–
miR-21	Induces tumor angiogenesis in vitro	PTEN	+
miR-24	Decreases endothelial cell proliferation	Sp1	–
miR-26a	Prevents endothelial cell apoptosis	TRPC6	+
miR-27a	Promotes EC angiogenesis in vitro	SEMA6A, Spry2, Dll4	+
miR-27b	Promotes EC angiogenesis in vitro	SEMA6A, Spry2, Dll4	+
miR-126	Promotes EC angiogenesis in vitro and in vivo	Spred-1, PIK3R2, VCAM-1	+
miR-150	Restores vascular barrier function	Ang2	+
miR-146a	Promotes senescence of endothelial cells	NOX4	–
miR-155	Promotes tumor angiogenesis	VHL	+
miR-221	Inhibits EC-mediated angiogenesis in vitro	c-kit, eNOS	–
miR-223	Prevents endothelial cell proliferation	β1 integrin, IGF-1R	–
miR-320	Inhibits diabetic angiogenesis in vitro	IGF-1	–

miR: microRNA, −: Negative regulation, +: Positive adjustment

### General characteristics of the patients with ARDS

A total of 101 patients admitted to the ICU of Zhongda Hospital Affiliated to Southeast University from January 2016 to September 2016; diagnosed ARDS were inspected. Ultimately, 44 patients were excluded (30 malignant tumor patients, six patients administered glucocorticoid in the past 6 months, five patients older than 90 years old and three pregnant women). Fifty-seven were included in the study: 43 cases in ARDSp group and 14 cases in ARDSexp group. Age, BMI, APACHE II score, SOFA score, lactic acid, 28-day mortality rate had no statistical difference (*P* > 0.05) between ARDSp and ARDSexp. General data of the 57 ARDS are listed in Table 2.

**Table 2:** General data comparison between ARDSp and ARDSexp

Variable	Total (*n* = 57)	ARDS_p_(1) (*n* = 43)	ARDS_exp_(2) (*n* = 14)	*P* value (1) versus (2)
*General condition*				
Age (years)	59.0 ± 17.5	56.6 ± 20.4	63.7 ± 12.6	0.13
Male* n* (%)	41 (71.9%)	30 (69.8%)	11 (78.6%)	0.52
BMI	23.9 ± 3.6	24.0 ± 3.8	23.6 ± 3.0	0.70
APACHE II score	21.3 ± 8.4	21.8 ± 8.5	20.0 ± 8.4	0.50
SOFA score	10.4 ± 4.9	10.4 ± 4.6	10.3 ± 5.7	0.93
28-day mortality	18 (31.6%)	14 (32.6%)	4 (22.2%)	1.00
*Basic diseases*				
COPD *n *(%)	1 (1.8%)	0 (0%)	1 (7.1%)	0.25
Hypertension *n *(%)	16 (28.1%)	13 (30.2%)	3 (21.4%)	0.77
CHD *n* (%)	8 (14.0%)	7 (16.3%)	1 (7.1%)	0.68
CVD *n* (%)	8 (14.0%)	8 (18.6%)	0 (0%)	0.19
DM *n* (%)	12 (21.1%)	10 (23.3%)	2 (14.3%)	0.74
HBD *n* (%)	7 (12.3%)	1 (2.3%)	6 (42.9%)	0.001
ISD *n* (%)	0 (%)	0 (%)	0 (%)	1.00
*ARDS etiology*				
PI *n *(%)	36 (63.2%)	36 (83.7%)	0 (0%)	< 0.001
Inhalation *n* (%)	3 (5.3%)	3 (7.0%)	0 (0%)	0.57
PC *n* (%)	4 (7.0%)	4 (9.5%)	0 (0%)	0.515
Sepsis *n* (%)	3 (5.3%)	0 (0%)	3 (20%)	0.016
Pancreatitis *n *(%)	4 (7.0%)	0 (0%)	4 (26.7%)	0.004
EPT *n* (%)	5 (8.8%)	0 (0%)	5 (33.3%)	0.001
Others *n* (%)	2 (3.5%)	0 (0%)	2 (14.3%)	0.057
*Organ dysfunction*				
Septic shock *n* (%)	22 (38.6%)	17 (39.5%)	5 (35.7%)	0.23
AKI *n* (%)	14 (24.6%)	10 (23.3%)	4 (28.6%)	0.97

*BMI* body mass index, *COPD* chronic obstructive pulmonary disease, *ARDS* acute respiratory distress syndrome, *AKI* acute kidney injury, *APACHE* acute physiology and chronic health evaluation, *SOFA* sequential organ failure assessment, *CHD* coronary heart disease, *CVD* cerebrovascular disease, *DM* diabetes mellitus, *HBD* hepatobiliary diseases, *ISD* immune system disease, *PI* pulmonary infection, *PC* pulmonary contusion, *EPT* extrapulmonary trauma

### Comparison of patient’s clinical condition indexes between ARDSp and ARDSexp

Indicators from clinical monitoring and laboratory detection were compared between ARDSp and ARDSexp. Oxygenation index (PO_2_/FiO_2_) in ARDSp was lower than that in ARDSexp (145 [119–203] vs. 206 [184–253], *P* = 0.012). Murray lung injury score in ARDSp was significantly higher than ARDSexp (2.7 [2–3.3] vs. 1.8 [1.3–2.4], *P* = 0.008). FiO_2_ and PEEP had no statistical difference between ARDSp and ARDSexp (*P* > 0.05). The proportion of ECMO, CRRT and invasive mechanical ventilation treatment had no statistical difference between ARDSp and ARDSexp (*P* > 0.05). Indexes related to infection and shock had no statistical difference between two groups (*P* > 0.05) (Table 3).

**Table 3:** Comparison of patient’s clinical condition indexes between ARDSp and ARDSexp

Variable	Total (*n* = 57)	ARDS_p_(1) (*n* = 43)	ARDS_exp_(2) (*n* = 14)	*P* value (1) versus (2)
*Lung injury severity*				
PH	7.4 [7.35–7.45]	7.41 [7.36–7.46]	7.37 [7.32–7.43]	0.26
FiO_2_	0.5 [0.4–0.6]	0.5 [0.4–0.6]	0.4 [0.4–0.5]	0.06
PEEP(cmH2O)	8 [5–12]	8 [5–12]	5 [5–12]	0.54
PO_2_/FiO_2_(mmHg)	165 [112–211]	145 [110–203]	206 [184–253]	0.012
Murray score	2.3 [1.7–3.1]	2.7 [2–3.3]	1.8 [1.3–2.4]	0.008
*Infection index*				
Leukocyte count	10.4 [6–16.7]	10.4 [6.4–14.5]	11.8 [5.5–18.3]	0.81
Platelet count	134 [90–188]	134 [107–195]	128 [49–184]	0.38
CRP	74 [25–128]	74 [31–121]	75 [15–141]	0.87
PCT	1.3 [0.2–12.9]	1. 0 [0.2–13.1]	1.86 [0.7–10.4]	0.60
*Shock index*				
HR	73 [63–121]	72 [63–120]	76 [63–125]	0.66
NE	5 [0–27.5]	4 [0–20]	5 [0–85]	0.21
Lactic acid	2.1 [1–3.1]	2.0 [0.9–2.9]	2.2[1.2–5.4]	0.11
*Organ supporting*				
IMV *n* (%)	40 (70.2%)	32 (74.4%)	8 (57.1%)	0.37
ECMO *n* (%)	12 (21.1%)	12 (27.9%)	0 (0%)	0.065
CRRT *n* (%)	10 (17.5%)	7 (16.3%)	3 (21.4%)	0.97

*PH* arterial blood pH value, *PO*_*2*_ arterial partial pressure of oxygen, *FiO*_*2*_ oxygen concentration, *PEEP* positive end expiratory pressure, *Murray score* lung injury score used for ARDS patients, *CRP* C reactive protein, *PCT* procalcitonin, *IMV* invasive mechanical ventilation, *ECMO* extracorporeal membrane oxygenation, *CRRT* continuous renal replacement therapy, *HR* heart rate, *NE* norepinephrine. *P* < 0.05 suggests statistical difference

### Comparison of plasma *MSC*-*VEC*-miRNAs levels between ARDSp and ARDSexp

**Fig. 1:**
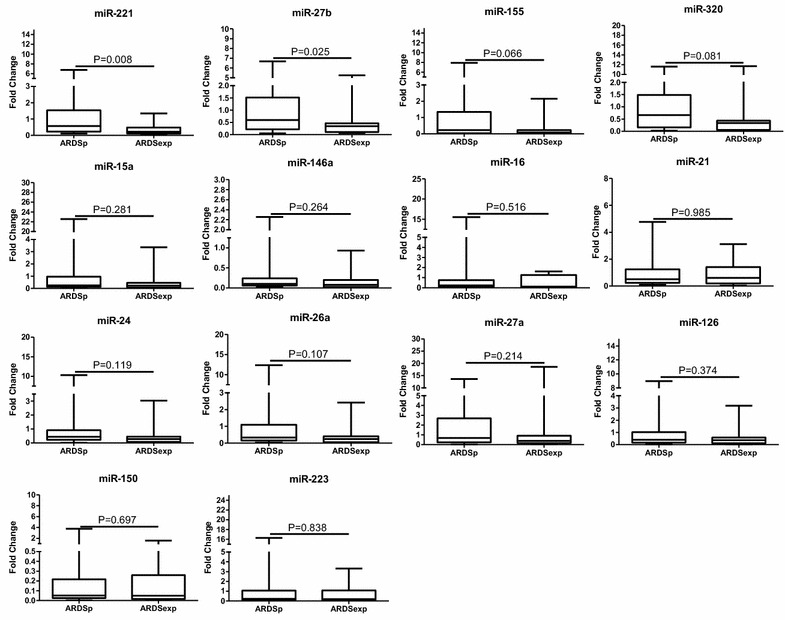
Comparison of *MSC*-*VEC*-miRNAs between ARDSp and ARDSexp. Data presented as a relative fold change between ARDSp and ARDSexp for each miRNA. Box plots are displayed where the horizontal bar represents the median, the box represents the IQR, and the whiskers represent the maximum and minimum values. Comparisons made by Mann–Whitney *U* test. *miRNA* microRNA, *IQR* interquartile range

Plasma miR-221 and miR-27b levels in ARDSexp group exhibited significantly lower than that in ARDSp group (0.22 [0.12–0.49] vs. 0.57 [0.22–1.57], *P* = 0.008), (0.34 [0.10–0.46] vs. 0.60 [0.20–1.46], *P* = 0.025). Other 12 kinds of plasma miRNAs levels between two groups showed no statistical difference. Plasma levels of *MSC*-*VEC*-miRNAs between ARDSp and ARDSexp are shown in Fig. 1.

### Comparison of plasma vWF, VCAM-1, IL10, TNFα concentration between ARDSp and ARDSexp

**Fig. 2:**
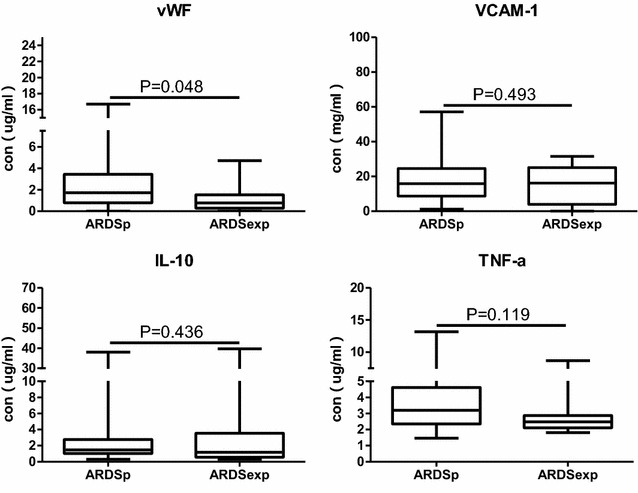
Comparison of plasma vWF, VCAM-1, IL10, TNFα concentration between ARDSp and ARDSexp. The concentration unit of vWF, IL10, TNFα is ug/ml. The concentration unit of VCAM-1 is mg/ml. Box plots are displayed where the horizontal bar represents the median, the box represents the IQR and the whiskers represent the maximum and minimum values. Comparisons made by Mann–Whitney *U* test

Plasma vWF concentration in ARDSexp group exhibited significantly lower than that in ARDSp group (0.77 [0.29–1.54] vs. 1.80 [0.95–3.51], *P* = 0.048). However, VCAM-1, IL10, TNFα concentration between two groups showed no statistical difference. Plasma concentration of VCAM-1, IL10, TNFα between ARDSp and ARDSexp is shown in Fig. 2.

### The correlation of plasma levels between miR-27b/miR-221 and vWF

**Fig. 3:**
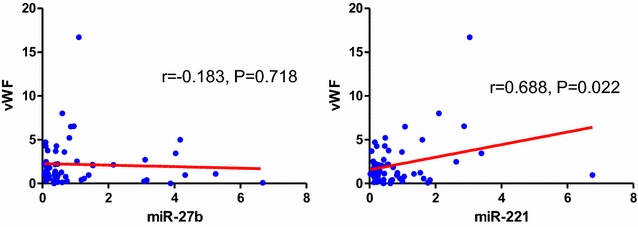
Correlation between vWF and miR-27b/miR-221

As plasma miR-27b/miR-221 and vWF levels were significant different between ARDSp and ARDSexp groups, we analyzed the correlation of plasma levels between miR-27b/miR-221 and vWF. We found significant positive correlation between miR-221 and vWF in plasma levels (*r* = 0.688, *P* = 0.022). However, there was no significant correlation between miR-27b and vWF in plasma levels (Fig. 3).

### Comparison of plasma patient’s clinical illness condition data between survival and non-survival group in ARDSp patients

APACHE II score, SOFA score, P/F, Murray score, CRP, Lactic acid were used as common indicators to evaluate ARDS patients’ clinical illness condition. This study showed that APACHE II score, SOFA score and lactic acid in survival group were significantly lower than that in non-survival group (APACHE II score: 18.7 ± 7.6 vs. 28.1 ± 7.6, *P*<0.001; SOFA score: 8.8 ± 4.1 vs. 14.0 ± 3.8, *P*<0.001; lactic acid: 1.7 [0.9–2.2] vs. 2.9 [1.2–3.3], *P* = 0.015) in ARDSp patients. P/F, Murray score and CRP between two groups showed no statistical difference (Table 4).

**Table 4:** Comparison of patient’s clinical illness condition data in ARDSp patients

Variable	Survival (*n* = 29)	Non-survival (*n* = 14)	*P* value
APACHE II score	18.7 ± 7.6	28.1 ± 7.6	< 0.001
SOFA score	8.8 ± 4.1	14.0 ± 3.8	< 0.001
P/F(mmHg)	150 [113–203]	130 [100–195]	0.39
Murray score	2.6 [2.0–3.0]	3.0 [2.3–3.7]	0.09
CRP	73.7 [31.0–111]	91.3 [33.3–121]	0.76
Lactic acid	1.7 [0.9–2.2]	2.9 [1.2–3.3]	0.015

### Comparison of plasma *MSC*-*VEC*-miRNAs levels between survival and non-survival group in ARDSp patients

**Fig. 4:**
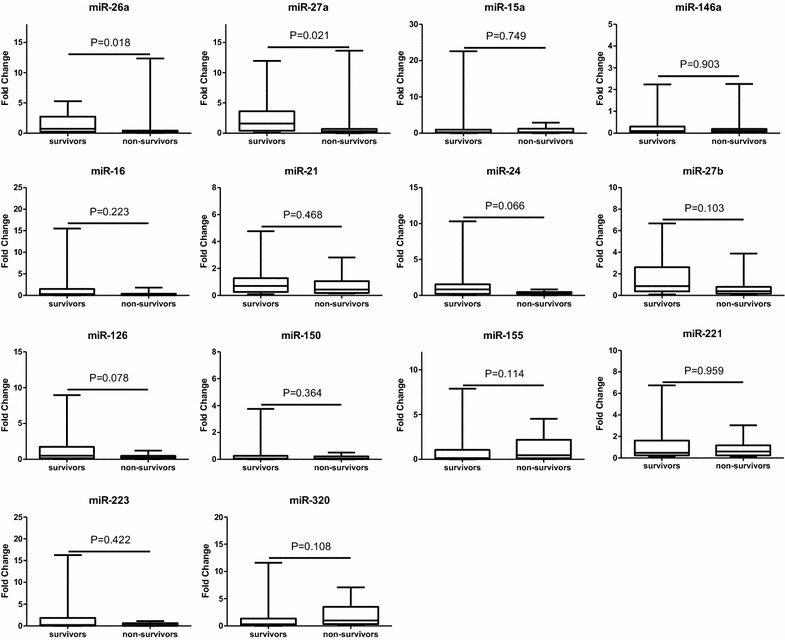
Comparison of *MSC*-*VEC*-miRNAs between survival group and death group in ARDSp patients. Data presented as a relative fold change between ARDSp and ARDSexp for each miRNA. Box plots are displayed where the horizontal bar represents the median, the box represents the IQR and the whiskers represent the maximum and minimum values. Comparisons made by Mann–Whitney U test. *miRNA* microRNA, *IQR* interquartile range

**Fig. 5:**
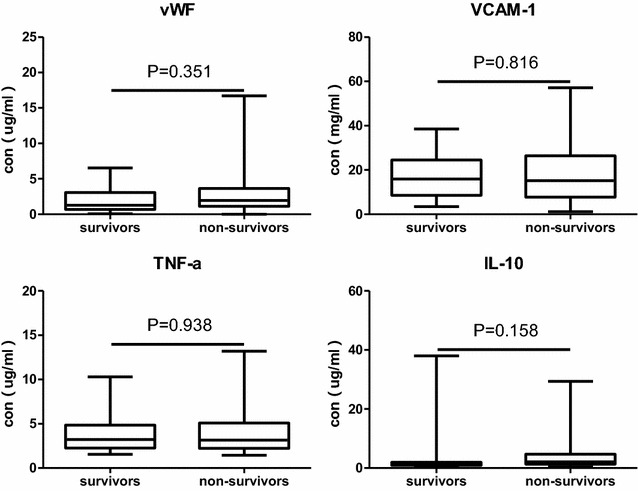
Comparison of plasma vWF, VCAM-1, IL10, TNFα concentration between survival group and death group in ARDSp patients. The concentration unit of vWF, IL10, TNFα is ug/ml. The concentration unit of VCAM-1 is mg/ml. Box plots are displayed where the horizontal bar represents the median, the box represents the IQR and the whiskers represent the maximum and minimum values. Comparisons made by Mann–Whitney U test

In our research, extrapulmonary ARDS was caused by sepsis, pancreatitis, extrapulmonary trauma etc. We just analyzed plasma *MSC*-*VEC*-miRNAs and vWF, VCAM-1, IL10, TNFα levels between 28 days survival and 28 days non-survival group in ARDSp patients in order to reduce the heterogeneity between patients. Plasma miR-26a and miR-27a levels in non-survival group exhibited significantly lower than that in survival group (miR-26a: 0.17 [0.08–0.20] vs. 0.69 [0.24–2.33] *P* = 0.018; miR-27a: 0.23 [0.16–0.58] vs. 1.45 [0.38–3.63], *P* = 0.021) in ARDSp patients. Other 12 kinds of miRNAs and vWF, VCAM-1, IL10, TNFα levels in plasma between two groups showed no statistical difference (Figs. 4, 5).

### The predictive value of miR-26a and miR-27a for prognosis of ARDSp patients

**Fig. 6:**
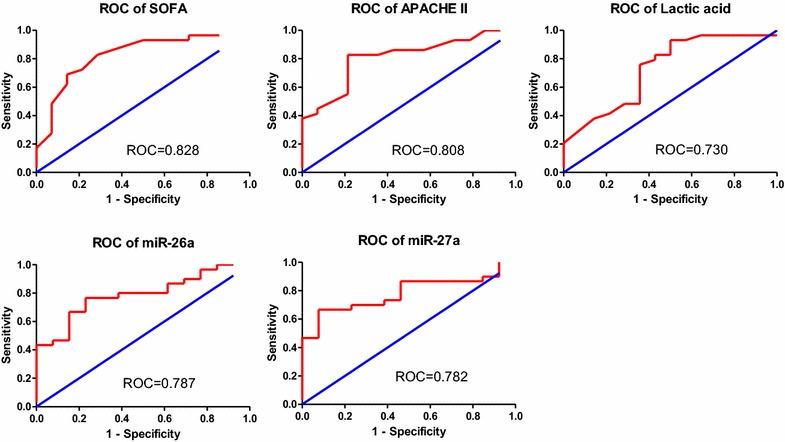
Receiver operating characteristic curve of SOFA score, APACHE II score, lactic acid value, miR-26a, miR-27a for predicting 28 days mortality in ARDSp patients

**Fig. 7:**
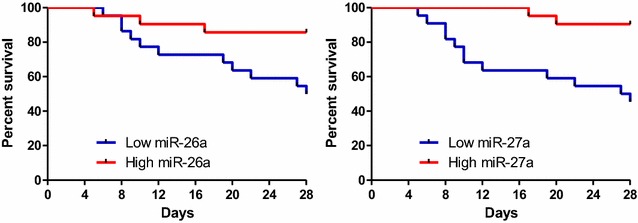
Probability of survival and subgroup analyses of the risk of death at 28 days

As APACHE II score, SOFA score, lactic acid, miR-26a and miR-27a were significantly different between non-survival and survival groups in ARDSp patients, ROC curves were drawn and the area under the curve (AUC) values for APACHE II score, SOFA score, lactic acid, miR-26a and miR-27a were, respectively, 0.808 (95%CI: 0.673–0.943), 0.828 (95%CI: 0.693–0.962), 0.782 (95%CI: 0.564–0.897), 0.787 (95%CI: 0.650–0.925), 0.782 (95%CI: 0.650–0.918) (Fig. 6). We also divided the patients into two groups according to median miR-26a or miR-27a value. Survival curve analysis showed that ARDSp patients with lower concentration of miR-26a/miR-27a had higher mortality (Fig. 7). Tables 5 and 6 show the results of the multivariate logistic regression analysis for the death of ARDSp patients. MiR-26a (OR: 1.483, 95% CI: 0.999–2.200, *P* = 0.050), miR-27a (OR: 1.425, 95% CI: 1.008–2.015, *P* = 0.045) were may independently associated with the death of ARDSp patients.

**Table 5:** Multivariable analysis (miR-26a included) of the death of ARDSp patients

	Odds ratio	95% CI	*P* value
(Intercept)	3931.707	–	0.004
Age	0.959	0.908–1.013	0.133
APACHE II score	0.791	0.679–0.921	0.003
miR-26a	1.483	0.999–2.200	0.050

**Table 6:** Multivariable analysis (miR-27a included) of the death of ARDSp patients

	Odds ratio	95% CI	*P* value
(Intercept)	1673.348	–	0.007
Age	0.964	0.914–1.017	0.185
APACHE II score	0.803	0.679–0.926	0.002
miR-27a	1.425	1.008–2.015	0.045

## Discussion

The results of this study demonstrate that the expression of plasma miR-221, miR-27b and endothelial markers vWF is significantly different between ARDSp and ARDSexp patients. Plasma miR-26a and miR-27a levels showed significantly different between non-survival group and survival group in ARDSp patients.

The characteristics of the enrolled patients in this study may impact research results. The ARDSp patients are more serious than the ARDSexp patients in the local lung injury and lung function lesion. The ARDSp patients owned higher Murray lung injury score and lower PO_2_/FiO_2_ than the ARDSexp patients and included all 12 patients received ECMO treatment. But indicators related to the overall illness condition, such as APACHE II scores, SOFA scores, blood lactate levels, doses of norepinephrine and the proportion of complicating sepsis, septic shock, AKI showed no statistical difference between ARDSp and ARDSexp patients. There was no difference in the 28-day mortality between the two groups, probably because the overall illness condition had no difference between the two groups. Therefore, the survival rate depends on overall illness severity or, say, the systematic condition of the whole organ rather than single organ lesions. We should pay attention to primary disease treatment and, meanwhile, systematic organ maintenance to prevent multiple organ dysfunction on critically ill patients.

In our study, pulmonary vascular endothelium lesion in ARDSp patients may be more serious than that in ARDSexp patients which embody in Murray lung injury score and PO_2_/FiO_2_. The result is in agreement with the previous research [24–26]. Previous studies show that miR-27b promotes vascular endothelial cell angiogenesis, yet miR-221 inhibits vascular endothelial cell-mediated angiogenesis. So, we deem ARDSp patients will express higher levels of miR-221 and, conversely, express reduced levels of miR-27b than ARDSexp patients. However, our research shows that plasma miR-221 and miR-27b levels in ARDSexp group exhibited significantly lower than that in ARDSp group which is inconsistent with expected results. We reviewed forepassed clinical researches and acquired contradictory results with each other. Significant increase in miR-27b expression was observed in the serum samples of patients with peripheral artery disease and arteriosclerosis obliterans when compared to the controls [27, 28]. Coskunpinar et al. [29] reported an increased plasma expression level of miR-221 in acute myocardial infarction compared with healthy controls. However, Tsai et al. presented that stroke patients and atherosclerosis subjects had significantly lower miR-221 serum levels than healthy controls [30]. These conclusions give us a hint that the expression of miRNAs is complex in different diseases originating from the similar pathological change.

Meanwhile, this research explored endothelial markers vWF, VCAM-1 and inflammatory cytokines IL10, TNFα. Plasma vWF concentration in ARDSexp group exhibited significantly lower than that in ARDSp group; however, plasma VCAM-1, IL10, TNFα concentration showed no statistical difference between two groups. As far as we know, endothelium can release vWF which forms additional links between the platelets’ glycoprotein and the collagen fibrils. To a certain extent, elevated vWF concentration reflected vascular endothelium lesion. But there was much controversy as to whether vWF could serve as a biomarker for ARDS. VWF is considered as in vivo and in vitro marker of endothelial injury in patients with ARDS [31]. It has previously been reported that high plasma level of vWF was associated with a greater risk of developing ARDS in sepsis patients and was associated with higher mortality in patients with established ARDS [31–34]. It also was reported that plasma levels of vWF did not appear to serve as useful markers for predicting ARDS in patients at risk and mortality in ARDS patients [35–37]. The vWF studies in ARDSp and ARDSexp are rare. Calfee et al. [38] reported that plasma vWF levels were significantly lower in ARDSp than that in ARDSexp which was not consistent with our result. It may be because patients in ARDSexp group were severer with higher APACHE III score and mortality in this study which was not consistent with our research, too. Upregulation of VCAM-1 in endothelial cells by cytokines partly occurs as a result of increased gene TNFα transcription. So, in our results, VCAM-1 and TNFα change in the same direction. In our study, leukocyte count, PCT, CRP showed no statistical difference between two groups, which is consistent with the change direction of IL10, TNFα.

Significant positive correlation between miR-221 and vWF in plasma levels was found in our study. Circulating is mostly released constitutively from endothelial storage organelles, Weibel–Palade bodies (WPBs) [39, 40]. WPBs are released from endothelial cells in response to a large number of agonists which include two distinct groups: those that act by elevating intracellular calcium ion (Ca^2+^) levels and those that act by raising cAMP levels in the cell [41–44]. Xiang et al. [45] identified that miR-24 and miR-335 targeted human vWF 3’UTR. Previous studies with regard to miR-221 regulating vWF production are absent. However, miR-221 increases free Ca^2+^ level of mast cells by PI3 K/Akt/PLCγ/Ca^2+^ signaling pathway [46]. MiR-221 may have the same regulatory role in vascular endothelial cells. The reasons of the positive correlation between miR-221 and vWF in plasma levels need to be studied further.

Because the etiology of extrapulmonary ARDS is diverse, we just analyzed plasma *MSC*-*VEC*-miRNAs and vWF, VCAM-1, IL10, TNFα levels between 28 days survival and 28 days non-survival group in ARDSp patients in order to reduce the heterogeneity between patients. In ARDSp patients, plasma miR-26a and miR-27a levels in non-survival group exhibited significant statistical differences. Plasma levels of miR-26a and miR-27a were lower in non-survival group, which might be because the two miRNAs were protective factors of vascular endothelial cell. APACHE II score, SOFA score, and lactic acid value showed significant statistical differences between two groups. Receiver operating characteristic curve (ROC curve) showed that SOFA score, APACHE II score, lactic acid value, miR-26a, miR-27a roughly equally predict the prognosis of ARDSp patients. Survival curve intuitively points out that plasma miR-26a and miR-27a levels were associated with mortality in ARDSp patients. So, miR-26a and miR-27a may be potential biomarkers for predicting the prognosis of ARDSp patients, the molecular mechanisms behind this which need to be further studied.

There are limitations in this study. Firstly, as stated above, our candidate miRNAs limited to the *MSC*-*VEC*-miRNAs, which is associated with MSC, has regulatory effects on VEC and has previously been studied in ARDS. The broader miRNA spectrum needs to be involved in future research. Secondly, this study is a clinical observational study, but not involved the molecular mechanism of miRNA regulation in cell. So, we cannot determine where the differential expression of plasma miR-221 and miR-27b come from and which results the difference contributes to. Thirdly, the sample size is relatively small which may have limited the power of statistical difference in this study.

In conclusion, ARDSp patients have higher Murray lung injury score and worse oxygenation index than ARDSexp patients in our study. Plasma miR-221, miR-27b and vWF levels in ARDSexp patients exhibited significantly lower than that in ARDSp patients. Significant positive correlation was found between miR-221 and vWF in plasma levels. In addition, we found plasma miR-26a and miR-27a levels in non-survival group exhibited significantly lower than that in survival group in ARDSp patients.

## Additional files

**Additional file 1: Table S1.** Summary of candidate miRNAs searched by articles.

**Additional file 2: Table S2.** Relevant references for candidate miRNAs.

## Abbreviations

ARDS: acute respiratory distress syndrome
MSC: mesenchymal stem cells
VEC: vascular endothelial cells
ARDSp: pulmonary ARDS
ARDSexp: extrapulmonary ARDS
miRNAs: microRNAs, miR microRNA
ICU: intensive care unit
vWF: von Willebrand factor
APACHE: acute physiology and chronic health evaluation
SOFA: sequential organ failure assessment
KDIGO: kidney disease improving global outcomes
ECMO: extracorporeal membrane oxygenation
CRRT: continuous renal replacement therapy
VCAM-1: vascular cell adhesion molecule 1
IL: interleukin
TNFα: tumor necrosis factor α
CRP: c-Reactive protein
PCT: procalcitonin
ROC: receiver operating characteristic
AUC: area under the curve
AKI: acute kidney injury
IQR: interquartile range
