# Supplementary material for: Plasma microRNAs levels are different between pulmonary and extrapulmonary ARDS patients: a clinical observational study
Source: Ann Intensive Care. 2018 Feb 13;8:23. doi: 10.1186/s13613-018-0370-1 (PMC5811418; doi:10.1186/s13613-018-0370-1)
Supplement: Supplementary file 1 — Additional file 1: Table S1. Summary of candidate miRNAs searched by articles. [file 13613_2018_370_MOESM1_ESM.docx]

Table S1 Summary of candidate miRNAs searched by articles

| **Numeber** | **Search methods** | **No. of papers** |
| --- | --- | --- |
| **#1** | ((Mesenchymal Stromal Cell[Title/Abstract]) OR "Mesenchymal Stromal Cells"[Mesh]) OR Mesenchymal Stem Cell[Title/Abstract] | 28060 |
| **#2** | ("Endothelial Cells"[Mesh]) OR ((((endothelial cell[Title/Abstract]) OR VEC[Title/Abstract]) OR PVEC[Title/Abstract]) OR PEC[Title/Abstract]) | 86723 |
| **#3** | ("Respiratory Distress Syndrome, Adult"[Mesh]) OR (((Acute Lung Injury[Title/Abstract]) OR ARDS[Title/Abstract]) OR respiratory distress syndrome[Title/Abstract]) | 37681 |
| **#4** | ("MicroRNAs"[Mesh]) OR (((microRNA*[Title/Abstract]) OR miRNA*[Title/Abstract]) OR miR*[Title/Abstract]) | 68997 |
| **#5** | #2 AND #4 = “miRNAs cluster 1” | 1192 |
| **#6** | “miRNAs cluster 1” AND #1 = “miRNAs cluster 2” |  |
| **#7** | “miRNAs cluster 2” AND #3 = “14 miRNA” = *MSC-VEC-*miRNA |  |
