# Supplementary material for: Plasma microRNAs levels are different between pulmonary and extrapulmonary ARDS patients: a clinical observational study
Source: Ann Intensive Care. 2018 Feb 13;8:23. doi: 10.1186/s13613-018-0370-1 (PMC5811418; doi:10.1186/s13613-018-0370-1)
Supplement: Supplementary file 2 — Additional file 2: Table S2. Relevant references for candidate miRNAs. [file 13613_2018_370_MOESM2_ESM.docx]

Table S2 Relevant references for candidate miRNAs

| **miRNAs** | **relating to VEC** | **relating to MSC** | **relating to ARDS** |
| --- | --- | --- | --- |
| miR-15a | [1-4] | [37,38] | [56] |
| miR-16 | [3,4] | [37,38] | [40,41] |
| miR-21 | [5-11] | [37,38,40] | [40,44,49,54,57] |
| miR-24 | [12,13] | [38] | [40,58] |
| miR-26a | [59] | [37,38] | [40,44,58] |
| miR-27a | [15] | [37,38] | [46,53,56,57] |
| miR-27b | [15-17] | [37,38] | [44,46] |
| miR-126 | [18-26] | [38,40] | [44,58] |
| miR-150 | [27] | [40] | [46] |
| miR-146a | [14] | [38] | [42,44,45,47,52,57] |
| miR-155 | [19,28] | [38] | [43,46,48,50,52,53,55,57] |
| miR-221 | [29-33] | [37,38] | [46] |
| miR-223 | [34,35] | [38,39] | [46,49] |
| miR-320 | [36] | [38] | [51] |

[1] P: 22692216, [2] P : 20445066, [3] P: 17205120, [4] P: 16166262,

[5] P: 24895913, [6] P: 24732886, [7] P: 20844033, [8] P: 21544242,

[9] P: 21347332, [10] P: 17478730, [11] P: 18971265, [12] P: 25920448,

[13] P: 24854275, [14] P: 21511256, [15] P: 22184411, [16] P: 22207734,

[17] P: 17540974, [18] P: 26659078, [19] P: 23353819, [20] P: 24014835,

[21] P: 23713864, [22] P: 22170610, [23] P: 18694566, [24] P: 18987025,

[25] P: 18227515, [26] P: 18694565, [27] P: 26743170, [28] P: 21310411,

[29] P: 16849646, [30] P: 17379831, [31] P: 22138289, [32] P: 19351599,

[33] P: 23409087, [34] P: 24307738, [35] P: 24044949, [36] P: 18986336,

[37] P: 25087724, [38] P: 26862575, [39] P: 26348153, [40] P: 22185353,

[41] P: 22940131, [42] P: 23848342, [43] P: 23514739, [44] P: 23124245,

[45] P: 24277697, [46] P: 23286498, [47] P: 24815778, [48] P: 24778118,

[49] P: 24736893, [50] P: 25553444, [51] P: 25972997, [52] P: 26137246,

[53] P: 26147972, [54] P: 26302186, [55] P: 26195546, [56] P: 26683209,

[57] P: 22659882, [58] P: 25070658, [59] P: 25801675

P PubMed Unique Identifier
